# Molecular Evolution of the RNA-Dependent RNA Polymerase and Capsid Genes of Human Norovirus Genotype GII.2 in Japan during 2004–2015

**DOI:** 10.3389/fmicb.2017.00705

**Published:** 2017-04-25

**Authors:** Fuminori Mizukoshi, Koo Nagasawa, Yen H. Doan, Kei Haga, Shima Yoshizumi, Yo Ueki, Michiyo Shinohara, Mariko Ishikawa, Naomi Sakon, Naoki Shigemoto, Reiko Okamoto-Nakagawa, Akie Ochi, Koichi Murakami, Akihide Ryo, Yoshiyuki Suzuki, Kazuhiko Katayama, Hirokazu Kimura

**Affiliations:** ^1^Department of Microbiology, Tochigi Prefectural Institute of Public Health and Environmental ScienceUtsunomiya-shi, Japan; ^2^Infectious Disease Surveillance Center, National Institute of Infectious DiseasesMusashimurayama-shi, Japan; ^3^Department of Virology II, National Institute of Infectious DiseasesMusashimurayama-shi, Japan; ^4^Laboratory of Viral infection I, Kitasato Institute for Life Sciences Graduate School of Infection Control Sciences, Kitasato UniversityMinato-ku, Japan; ^5^Department of Infectious Diseases, Hokkaido Institute of Public HealthSapporo-shi, Japan; ^6^Department of Microbiology, Miyagi Prefectural Institute of Public Health and EnvironmentSendai-shi, Japan; ^7^Virus Division, Saitama Institute of Public HealthHiki-gun, Japan; ^8^Division of Virology, Kawasaki City Institute for Public HealthKawasaki-shi, Japan; ^9^Department of Microbiology, Osaka Prefectural Institute of Public HealthOsaka-shi, Japan; ^10^Hiroshima Prefectural Technology Research Institute Public Health and Environment CenterHiroshima-shi, Japan; ^11^Department of Health Science, Yamaguchi Prefectural Institute of Public Health and EnvironmentYamaguchi-shi, Japan; ^12^Department of Microbiology, Ehime Prefectural Institute of Public Health and Environmental ScienceMatsuyama-shi, Japan; ^13^Department of Microbiology, Yokohama City University Graduate School of MedicineYokohama-shi, Japan; ^14^Division of Biological Science, Nagoya City UniversityNagoya-shi, Japan

**Keywords:** norovirus, capsid, RNA-dependent RNA polymerase, molecular epidemiology, phylogeny, molecular evolution

## Abstract

The RNA-dependent RNA polymerase (*RdRp*) and capsid (*VP1*) genes of 51 GII.2 human norovirus (HuNoV) strains collected during the period of 2004–2015 in Japan were analyzed. Full-length analyses of the genes were performed using next-generation sequencing. Based on the gene sequences, we constructed the time-scale evolutionary trees by Bayesian Markov chain Monte Carlo methods. Time-scale phylogenies showed that the *RdRp* and *VP1* genes evolved uniquely and independently. Four genotypes of GII.2 (major types: GII.P2-GII.2 and GII.P16-GII.2) were detected. A common ancestor of the GII.2 *VP1* gene existed until about 1956. The evolutionary rates of the genes were high (over 10^−3^ substitutions/site/year). Moreover, the *VP1* gene evolution may depend on the *RdRp* gene. Based on these results, we hypothesized that transfer of the *RdRp* gene accelerated the *VP1* gene evolution of HuNoV genotype GII.2. Consequently, recombination between ORF1 (polymerase) and ORF2 (capsid) might promote changes of GII.2 antigenicity.

## Introduction

Human norovirus (HuNoV) is a major causative agent of gastroenteritis in humans (Green, 2013). The HuNoV genogroup II (GII), in particular, is frequently detected in outbreaks. The HuNoV GII strains can be classified into 22 genotypes (Kroneman et al., 2013). Moreover, the most worldwide prevalent HuNoV GII genotypes belong to GII genotype 2 (GII.2), GII.3, GII.4, GII.6, and GII.17 (Centers for Disease Control and Prevention. CaliciNet Data [cited 2016])^1^. Since national surveillance began, nearly 3 million cases of NoV gastroenteritis have been recorded, and Japan was experiencing its second most serious norovirus outbreak during November 2016 to February 2017 (National Institute of Infectious Diseases. Japan. Infectious Gastroenteritis. [cited 4th April 2017, in Japanese])^2^ Importantly, HuNoV GII.2 emerged as a major cause of this outbreak in Japan, although the GII.4 strains were the most prevalent genotype during the past 10 years (National Institute of Infectious Diseases. Japan. Flash report of norovirus in Japan [cited 4th April 2017, in Japanese])^3^.

Very recent studies suggested that the evolutionary patterns of human and animal NoV genotypes are distinct (Kobayashi et al., 2015, 2016). Although all viral proteins may act as antigens, the HuNoV VP1 protein is also involved in viral infection. Furthermore, HuNoV frequently experiences recombination at the ORF1/ORF2 junction, resulting in new chimera viruses with different types of the RNA-dependent RNA polymerase (*RdRp*) genes and capsid (*VP1*) genes. Most studies have focused on the molecular evolution of HuNoV GII.4. Only a few examined that gene in other HuNoV genotypes, including GII.2. To gain insight into this process, we examined the molecular evolution of the GII.2 *RdRp* and *VP1* genes, including chimera viruses, based on the full genome analyses of those detected in Japan over a period of 10 years (2004–2015 seasons).

## Materials and methods

To investigate the molecular evolution of the HuNoV *VP1* and *RdRp* genes, 950 stool specimens were collected from various areas (13 prefectures) of Japan during the 2004–2015 seasons. These samples were obtained from patients with acute gastroenteritis due to HuNoV infections, in compliance with the Food Sanitation Law and the Law Concerning the Prevention of Infections and Medical Care for Patients of Infections of Japan. The personal data related to these samples were anonymized. RNA was extracted from 10% PBS suspensions of the specimens, and the HuNoV genomes were comprehensively analyzed by next-generation sequencing as described (Matsushima et al., 2015). Of 950 samples, the complete genome sequences of 538 strains were obtained (a success rate of 57%). Next, HuNoV genotypes were confirmed with the Norovirus Typing Tool (Version1.0), based on the nucleotide sequences of *RdRp* and *VP1* genes as described by Kroneman et al. (2011). GII.2 strains were selected from these all genotyped strains, and then a few of strains having the undetermined base sequences (e.g., N, Y, R, and V) were omitted. Finally, 51 GII.2 strains were used for evolutionary analyses for the present study (Supplementary Table 1). The obtained nucleotide sequences for the GII.2 strains were deposited in GenBank under the accession numbers LC209431 to LC209481.

Time-scale evolutionary analyses were performed using the Bayesian Markov Chain Monte Carlo method (MCMC) with the BEAST package v1.8.3 (Drummond and Rambaut, 2007) and Tracer^4^ as a demographic model. Substitution models were calculated with Kakusan4 (Tanabe, 2011). The substitution model for the *VP1* or the *RdRp* gene was the GTR-Γ or GTR-Γ invariant model, respectively. Based on Akaike's Information Criterion for MCMC values, we used the random local clock as a clock model, and used the logistic growth model (*VP1* gene) or the constant size model (*RdRp* gene) as a tree model. Convergence was evaluated with an effective sample size (acceptable more than 200). The MCMC chain length was 3 × 10^8^ steps with sampling every 1,000 steps for the MCMC tree of the *VP1* gene. To exactly estimate the evolutionary rates and topologies of the MCMC tree of the *RdRp* gene, we bound two independent data of the MCMC chains^5^. The MCMC chain length was 2 × 10^8^ steps and 4 × 10^8^ with sampling every 5,000 steps for the MCMC tree of the *RdRp* gene. Statistical analyses were performed with the Welch's *t*-test in Excel 2013.

## Results and discussion

### Distribution of GII.2 genotype during the 2004–2015 seasons

**Figure 1:**
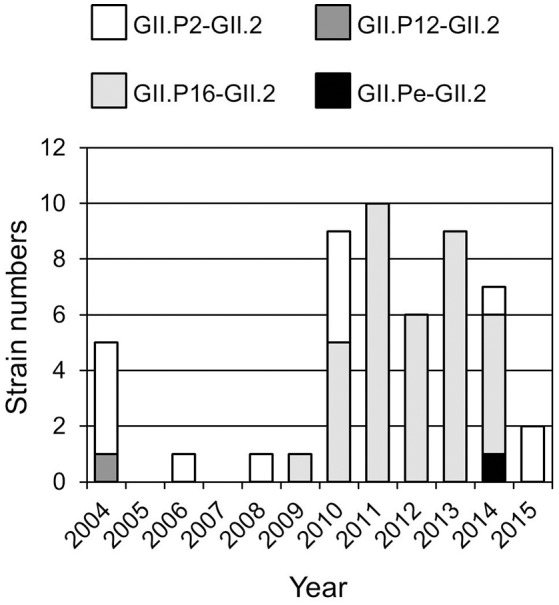
**Distribution of GII.2 genotype during the investigation periods (2004–2015 seasons)**.

Four genotypes of the GII.2 strains, including GII.P2-GII.2 (13 strains), GII.Pe-GII.2 (one strain), GII.P12-GII.2 (one strain), and GII.P16-GII.2 (36 strains), were determined by the Norovirus Typing Tool (Figure 1). Of them, GII.P16-GII.2 strains were the most prevalent genotype after 2009. The single GII.P12-GII.2 and GII.Pe-GII.2 strains were detected in 2004 and 2014 respectively. The GII.P2-GII.2 strains were detected throughout the investigation periods.

### Phylogenetic analysis and evolutionary rates of *VP1* and *RdRp* virus genes

**Figure 2:**
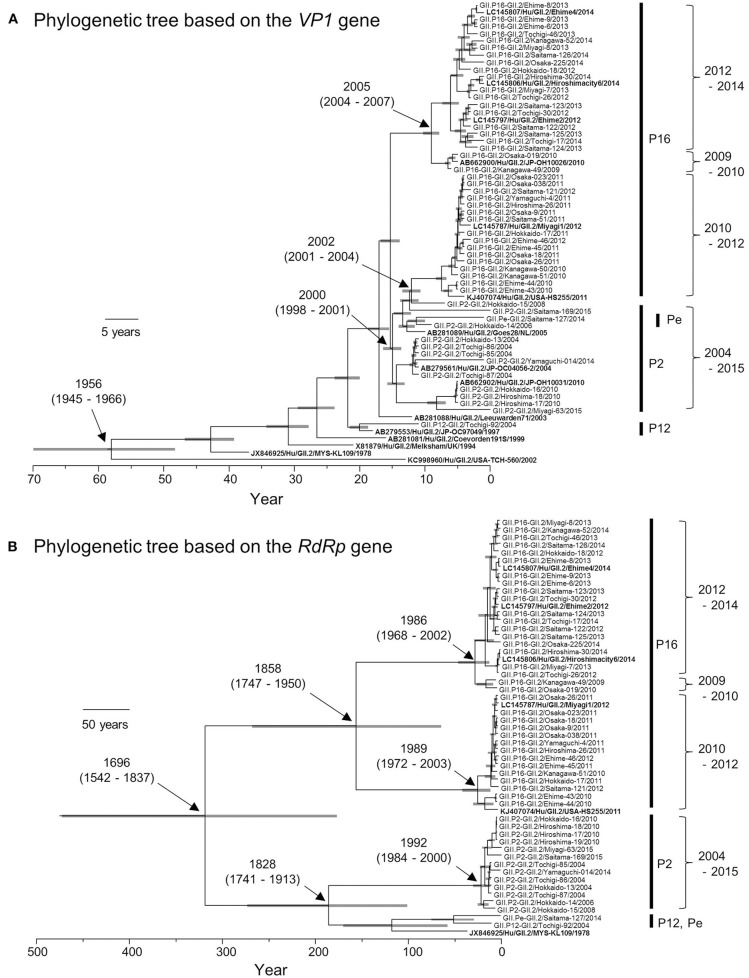
**Phylogenetic trees of *VP1* (A)** and *RdRp* **(B)** genes of the genotype GII.2 constructed by the Bayesian MCMC method. We analyzed *VP1* gene of 50 strains, and *RdRp* gene of 49 strains, excluding 100%—matched homologous strains. Reference strains in these trees were indicated in bold letters. Gray bar shows 95% HPD. The scale bar represents actual time (year).

Based on the *VP1* gene sequences, we constructed a time-scale evolutionary tree (Figure 2A). The phylogeny of the *VP1* gene showed that GII.2 strains could be divided according to the type of *RdRp* gene. GII.P16-GII.2 could be subdivided into three clusters of strains in 2009–2010, 2010–2012, and 2012–2014. In addition, the phylogenetic divergence of the GII.P16-GII.2 strains might be wider than that of the GII.P2-GII.2 strains. The tree shows that the most recent common ancestor (MRCA) of the present GII.2 strains appeared in 1956 (mean ± 95% highest posterior densities [HPD]: 1945–1966). Subsequently, GII.P2-GII.2 virus strain emerged in 2000 (mean ± 95% HPD: 1998–2001). Moreover, the GII.P16-GII.2 strains detected in 2010–2012 diverged from a common ancestor of the GII.P2-GII.2 strains at 2002 (mean ± 95% HPD: 2001–2004). The GII.P16-GII.2 strains detected in 2009–2010 and 2012–2014 diverged at 2005 (mean ± 95% HPD: 2004–2007). The evolutionary rate of these *VP1* genes was 2.987 × 10^−3^ substitutions/site/year (mean ± 95% HPD: 2.496–3.486 × 10^−3^ substitutions/site/year).

We also constructed a time-scale evolutionary tree of the *RdRp* gene (Figure 2B). The tree shows that the MRCA of *RdRp* of the present GII.2 strains was in the year 1696 (mean ± 95% HPD: 1542–1837). The common ancestor of the GII.P16-GII.2 strains diverged in 1858 (mean ± 95% HPD: 1747–1950) and formed two clusters. Moreover, the GII.P16-GII.2 strains detected in 2010–2012 diverged at 1989 (mean ± 95% HPD: 1972–2003), whereas the GII.P16-GII.2 strains detected in 2009–2010 and 2012–2014 diverged at 1986 (mean ± 95% HPD: 1968–2002). The common ancestor of the GII.P2-GII.2, GII.P12-GII.2, and GII.Pe-GII.2 diverged in 1828 (mean ± 95% HPD: 1741–1913). The *RdRp* gene of GII.P2-GII.2 diverged in 1992 (mean ± 95% HPD: 1984–2000). The evolutionary rate of these *RdRp* genes was 1.314 × 10^−3^ substitutions/site/year (mean ± 95% HPD: 0.698–1.95 × 10^−3^ substitutions/site/year).

Next, we compared the evolutionary rates of the GII.P16-GII.2 and GII.P2-GII.2 strains. To gain statistical significance, we collected the nucleotide sequences of the GII.P2-GII.2 strains (25 strains) from GenBank, but we could not collect a sufficient number of the GII.P12-GII.2 and GII.Pe-GII.2 sequences from the GenBank to reach statistical significance. The evolutionary rate of GII.P16-GII.2 (1.838 × 10^−3^ substitutions/site/year; mean ± 95% HPD: 1.237–2.456 × 10^−3^ substitutions/site/year) was greater than that of GII.P2-GII.2 (1.712 × 10^−3^ substitutions/site/year; mean ± 95% HPD: 0.957–2.41 × 10^−3^ substitutions/site/year) (*p* = 7.891 × 10^−135^).

A previous report suggested that the evolution of *VP1* may be influenced by the activities of *RdRp* (Bull et al., 2010). Collectively, our bioinformatics data also showed that the evolution of the GII.2 *VP1* gene was accelerated by a recombination of ORF1, including the *RdRp* gene. However, additional *in vitro* studies regarding the mutation rates of *RdRp* of the GII.P2 and GII.P16 may be needed to clarify the hypothesis of the relationships between and *VP1* and *RdRp* genes in this study. Furthermore, GII.2 variant strains were detected in the present season (2016/17 season), and thus, further genetic studies may be needed to prove this hypothesis.

## Conclusions

Here we report the molecular evolution of the *VP1* and *RdRp* genes in HuNoV GII.2. Our main findings and hypothesis are as follows. (1) Four genotypes of GII.2 (GII.P2-GII.2, GII.P16-GII.2, GII.P12-GII.2, and GII.Pe-GII.2) were detected in Japan in 2004–2015. (2) A common ancestor of the current GII.2 virus strains circulated around 1956. (3) *VP1* gene evolution seems to depend on the *RdRp* gene. The *VP1* gene in a prevalent HuNoV genotype GII.2 might evolve uniquely by transfer of the *RdRp* gene.

## Ethics statement

This study protocol was approved by the National Institute of Infectious Diseases Ethics Committee (No. 532).

## Author contributions

FM designed and performed the research, analyzed the data and wrote the manuscript. KN, YD, and KH performed the research and analyzed the data. FM, SY, YU, MS, MI, NS (Sakon), NS (Shigemoto), RO, and AO contributed samples and analyzed the data; and HK and KK designed and supervised the research, analyzed the data, and wrote the manuscript. All authors contributed, read, and approved the manuscript.

## Funding

This work was partly supported by a commissioned project for Research on Emerging and Re-emerging Infectious Diseases from Japan Agency for Medical Research and Development, AMED.

### Conflict of interest statement

The authors declare that the research was conducted in the absence of any commercial or financial relationships that could be construed as a potential conflict of interest.

## Footnotes

^1^http://www.cdc.gov/norovirus/reporting/calicinet/data.html

^2^http://www.nih.go.jp/niid/ja/10/2096-weeklygraph/1647-04gastro.html

^3^http://www.nih.go.jp/niid/ja/iasr-noro.html

^4^http://tree.bio.ed.ac.uk/software/tracer/

^5^http://beast.bio.ed.ac.uk/LogCombiner
