# Supplementary material for: Molecular Evolution of the RNA-Dependent RNA Polymerase and Capsid Genes of Human Norovirus Genotype GII.2 in Japan during 2004–2015
Source: Front Microbiol. 2017 Apr 25;8:705. doi: 10.3389/fmicb.2017.00705 (PMC5403926; doi:10.3389/fmicb.2017.00705)
Supplement: Supplementary file 1 [file Table1.DOCX]

Supplementary Material

Molecular Evolution of the RNA-Dependent RNA Polymerase and Capsid Genes in Human Norovirus Genotype GII.2 in Japan during 2004–2015

Fuminori Mizukoshi^1^, Koo Nagasawa^2^, Yen Hai Doan^3^, Kei Haga^4^, Shima Yoshizumi^5^, Yo Ueki^6^, Michiyo Shinohara^7^, Mariko Ishikawa^8^, Naomi Sakon^9^, Naoki Shigemoto^10^, Reiko Okamoto-Nakagawa^11^, Akie Ochi^12^, Koichi Murakami^2^, Akihide Ryo^13^, Yoshiyuki Suzuki^14^, Kazuhiko Katayama^4*^, Hirokazu Kimura^2, 13*^

*** Correspondence:** Hirokazu Kimura: kimhiro@nih.go.jp, Kazuhiko Katayama: katayama@lisci.kitasato-u.ac.jp

# Supplementary Table 1. Data of the present strains.

| Strain name | Genotype | Place of detection | Year | Accession Number |
| --- | --- | --- | --- | --- |
| GII.P12-GII.2/Tochigi-92/2004 | GII.P12-GII.2 | Tochigi | 2004 | LC209435 |
| GII.P2-GII.2/Hokkaido-13/2004 | GII.P2-GII.2 | Hokkaido | 2004 | LC209464 |
| GII.P2-GII.2/Tochigi-85/2004 | GII.P2-GII.2 | Tochigi | 2004 | LC209436 |
| GII.P2-GII.2/Tochigi-87/2004 | GII.P2-GII.2 | Tochigi | 2004 | LC209437 |
| GII.P2-GII.2/Tochigi-86/2004 | GII.P2-GII.2 | Tochigi | 2004 | LC209438 |
| GII.P2-GII.2/Hokkaido-14/2006 | GII.P2-GII.2 | Hokkaido | 2006 | LC209462 |
| GII.P2-GII.2/Hokkaido-15/2008 | GII.P2-GII.2 | Hokkaido | 2008 | LC209463 |
| GII.P16-GII.2/Kanagawa-49/2009 | GII.P16-GII.2 | Kanagawa | 2009 | LC209461 |
| GII.P16-GII.2/Ehime-44/2010 | GII.P16-GII.2 | Ehime | 2010 | LC209480 |
| GII.P16-GII.2/Ehime-43/2010 | GII.P16-GII.2 | Ehime | 2010 | LC209481 |
| GII.P16-GII.2/Kanagawa-51/2010 | GII.P16-GII.2 | Kanagawa | 2010 | LC209459 |
| GII.P16-GII.2/Kanagawa-50/2010 | GII.P16-GII.2 | Kanagawa | 2010 | LC209460 |
| GII.P16-GII.2/Osaka-019/2010 | GII.P16-GII.2 | Osaka | 2010 | LC209454 |
| GII.P2-GII.2/Hiroshima-18/2010 | GII.P2-GII.2 | Hiroshima | 2010 | LC209472 |
| GII.P2-GII.2/Hiroshima-19/2010 | GII.P2-GII.2 | Hiroshima | 2010 | LC209473 |

# Supplementary Table 1. Data of the present strains (Continued).

| Strain name | Genotype | Place of detection | Year | Accession Number |
| --- | --- | --- | --- | --- |
| GII.P2-GII.2/Hiroshima-17/2010 | GII.P2-GII.2 | Hiroshima | 2010 | LC209474 |
| GII.P2-GII.2/Hokkaido-16/2010 | GII.P2-GII.2 | Hokkaido | 2010 | LC209465 |
| GII.P16-GII.2/Ehime-45/2011 | GII.P16-GII.2 | Ehime | 2011 | LC209479 |
| GII.P16-GII.2/Hiroshima-26/2011 | GII.P16-GII.2 | Hiroshima | 2011 | LC209471 |
| GII.P16-GII.2/Hokkaido-17/2011 | GII.P16-GII.2 | Hokkaido | 2011 | LC209467 |
| GII.P16-GII.2/Osaka-9/2011 | GII.P16-GII.2 | Osaka | 2011 | LC209448 |
| GII.P16-GII.2/Osaka-26/2011 | GII.P16-GII.2 | Osaka | 2011 | LC209449 |
| GII.P16-GII.2/Osaka-18/2011 | GII.P16-GII.2 | Osaka | 2011 | LC209451 |
| GII.P16-GII.2/Osaka-038/2011 | GII.P16-GII.2 | Osaka | 2011 | LC209452 |
| GII.P16-GII.2/Osaka-023/2011 | GII.P16-GII.2 | Osaka | 2011 | LC209453 |
| GII.P16-GII.2/Saitama-51/2011 | GII.P16-GII.2 | Saitama | 2011 | LC209447 |
| GII.P16-GII.2/Yamaguchi-4/2011 | GII.P16-GII.2 | Yamaguchi | 2011 | LC209468 |
| GII.P16-GII.2/Ehime-46/2012 | GII.P16-GII.2 | Ehime | 2012 | LC209478 |
| GII.P16-GII.2/Hokkaido-18/2012 | GII.P16-GII.2 | Hokkaido | 2012 | LC209466 |
| GII.P16-GII.2/Saitama-122/2012 | GII.P16-GII.2 | Saitama | 2012 | LC209445 |
| GII.P16-GII.2/Saitama-121/2012 | GII.P16-GII.2 | Saitama | 2012 | LC209446 |
| GII.P16-GII.2/Tochigi-30/2012 | GII.P16-GII.2 | Tochigi | 2012 | LC209432 |
| GII.P16-GII.2/Tochigi-26/2012 | GII.P16-GII.2 | Tochigi | 2012 | LC209433 |
| GII.P16-GII.2/Ehime-9/2013 | GII.P16-GII.2 | Ehime | 2013 | LC209475 |
| GII.P16-GII.2/Ehime-8/2013 | GII.P16-GII.2 | Ehime | 2013 | LC209476 |
| GII.P16-GII.2/Ehime-6/2013 | GII.P16-GII.2 | Ehime | 2013 | LC209477 |
| GII.P16-GII.2/Miyagi-8/2013 | GII.P16-GII.2 | Miyagi | 2013 | LC209455 |
| GII.P16-GII.2/Miyagi-7/2013 | GII.P16-GII.2 | Miyagi | 2013 | LC209456 |
| GII.P16-GII.2/Saitama-125/2013 | GII.P16-GII.2 | Saitama | 2013 | LC209442 |
| GII.P16-GII.2/Saitama-124/2013 | GII.P16-GII.2 | Saitama | 2013 | LC209443 |
| GII.P16-GII.2/Saitama-123/2013 | GII.P16-GII.2 | Saitama | 2013 | LC209444 |

# Supplementary Table 1. Data of the present strains (Continued).

| Strain name | Genotype | Place of detection | Year | Accession Number |
| --- | --- | --- | --- | --- |
| GII.P16-GII.2/Tochigi-46/2013 | GII.P16-GII.2 | Tochigi | 2013 | LC209431 |
| GII.P16-GII.2/Hiroshima-30/2014 | GII.P16-GII.2 | Hiroshima | 2014 | LC209470 |
| GII.P16-GII.2/Kanagawa-52/2014 | GII.P16-GII.2 | Kanagawa | 2014 | LC209458 |
| GII.P16-GII.2/Osaka-225/2014 | GII.P16-GII.2 | Osaka | 2014 | LC209450 |
| GII.P16-GII.2/Saitama-126/2014 | GII.P16-GII.2 | Saitama | 2014 | LC209441 |
| GII.P16-GII.2/Tochigi-17/2014 | GII.P16-GII.2 | Tochigi | 2014 | LC209434 |
| GII.P2-GII.2/Yamaguchi-014/2014 | GII.P2-GII.2 | Yamaguchi | 2014 | LC209469 |
| GII.Pe-GII.2/Saitama-127/2014 | GII.Pe-GII.2 | Saitama | 2014 | LC209439 |
| GII.P2-GII.2/Miyagi-63/2015 | GII.P2-GII.2 | Miyagi | 2015 | LC209457 |
| GII.P2-GII.2/Saitama-169/2015 | GII.P2-GII.2 | Saitama | 2015 | LC209440 |
